# A Retrospective Evaluation of the Laboratory Findings of Dermatology Patients Whose Biotin Levels Were Checked

**DOI:** 10.7759/cureus.41482

**Published:** 2023-07-06

**Authors:** Hüsna Güder, Pınar Eker

**Affiliations:** 1 Department of Dermatology, Maltepe University Faculty of Medicine, İstanbul, TUR; 2 Department of Biochemistry, Maltepe University Faculty of Medicine, İstanbul, TUR

**Keywords:** laboratory findings, skin conditions, vitamins, triglyceride, biotin

## Abstract

Objective

Biotin is widely known to be beneficial for the hair, nails, and skin, but there are only a few studies on biotin. We evaluated whether there is a relationship between biotin levels and age, gender, and frequently observed laboratory findings. We also evaluated biotin levels according to the reason for checking biotin levels.

Methods

One hundred five patients applied to the dermatology outpatient clinic and had their biotin levels checked. Patient files were retrospectively analyzed.

Results

There were a weak positive (r=0.207) relationship between biotin levels and basophil count, a weak positive (r=0.201) relationship between biotin levels and creatinine, and a weak positive (r=0.314) relationship between biotin levels and cholesterol/triglyceride ratio. There were a weak negative (r=-0.216) relationship between biotin levels and mean platelet volume (MPV) and a moderately negative (r=-0.315) relationship between biotin levels and triglyceride levels.

Conclusion

Biotin levels do not significantly differ with gender but increase with age. Although a weak correlation was detected between hemogram parameters and biotin levels with basophil percentage and mean platelet volume values, biotin did not significantly change hemogram parameters. The relationship between biotin levels and triglyceride levels was the most critical finding of our study. We recommend examining biotin levels in the patients with high triglyceride levels. When we encounter dermatological side effects related to the use of epidermal growth factor receptor tyrosine kinase inhibitors, we recommend evaluating biotin levels. We recommend that biotin supplementation be made only in the patients with deficiencies and that biotin levels be measured in the follow-up.

## Introduction

Biotin is a water-soluble B-complex vitamin, also known as vitamin H or vitamin B7. Biotin is a coenzyme for carboxylases involved in many metabolic reactions. It plays a role in fatty acid synthesis, branched-chain amino acid catabolism, and gluconeogenesis [1]. It is known that biotin deficiency is mostly seen in biotinidase enzyme deficiency and raw egg consumption. Biotin deficiency may also occur in patients with inflammatory bowel disease and prolonged broad-spectrum antibiotic use, antiepileptic use, and isotretinoin use; alcoholics; smokers; patients receiving parenteral nutrition and partial gastrectomy; or patients with other achlorhydria [1,2].

Biotin deficiency can manifest through various symptoms, including hair loss, conjunctivitis, and red scaly dermatitis around the eyes, nose, and mouth. Other potential signs of biotin deficiency include depression, hallucinations, and numbness in the extremities. Inherited disorders related to biotin deficiency can further impact immune system function, leading to an increased vulnerability to bacterial and fungal infection [1-3].

Biotin is widely known to be beneficial for the hair, nails, and skin, but there are only a few studies on the role of biotin in skin diseases. In dermatology, it is suggested that biotin may play a role in hair loss, brittle nails, seborrheic dermatitis, and the reduction of xerosis in patients using isotretinoin [4].

We examined the relationship between biotin levels and frequently performed laboratory tests on the patients who underwent biotin examination in the dermatology outpatient clinic. In addition, we aimed to evaluate biotin status in some dermatological diseases and to investigate whether there is a relationship between biotin levels and hemogram, glucose, blood urea nitrogen (BUN), creatinine, aspartate aminotransferase (AST), alanine aminotransferase (ALT), cholesterol, triglyceride, vitamin, and mineral levels.

## Materials and methods

The study employed a retrospective cohort design to investigate the biotin levels of the patients attending the dermatology outpatient clinic. The inclusion criteria consisted of the patients whose biotin levels were checked between January 1, 2020, and January 1, 2023. Biotin examinations were conducted as a part of routine investigations for various dermatological conditions, without specifically targeting biotin deficiency.

A total of 105 patients were identified for inclusion in the study based on their biotin-level assessments. Patient information such as age, gender, comorbidities, and medication use was recorded. The prevalence of biotin deficiency is expected to vary across different age groups due to variations in dietary habits, physiological changes, metabolism, and overall health status. To investigate this further, we employed stratification by age, dividing the patients into three distinct categories: individuals under 18, those between 18 and 50, and those older than 50.

An evaluation of biotin levels has been performed in some situations that may be associated with various dermatological conditions. Biotin, along with other laboratory tests (such as complete blood count {CBC}, fasting glucose, blood urea nitrogen {BUN}, creatinine, aspartate aminotransferase {AST}, alanine aminotransferase {ALT}, cholesterol, triglyceride, vitamin B12, folic acid, ferritin, 25-OH vitamin D, zinc, and magnesium levels), were checked for the patients with acne scars, cold-induced hand dermatitis, seborrheic dermatitis, telogen effluvium, xerosis cutis, keloids, psoriasis, keratosis pilaris, alopecia areata, dyshidrotic eczema, rosacea, urticaria, wart, and vitiligo.

Complete blood cell parameters were performed using specific analyzers: Cell-Dyn 4,000 Ruby for CBC, Architect c8000 for clinical chemistry tests, and Architect i1000SR for chemiluminescence tests. All analyzers used in the study were manufactured by Abbott Laboratories, located in North Chicago, Illinois, USA. Biotin levels were measured in ng/L using the enzyme-linked immunosorbent assay (ELISA) method with an Alisei automated analyzer. The reference values for biotin levels were categorized as deficiency (<200 ng/L), suboptimal (200-400 ng/L), and optimal (>400 ng/L) [3].

The study retrospectively analyzed the prevalence of biotin deficiency among the patients and examined the relationship between biotin levels and other laboratory parameters based on the reason for the biotin examination.

Ethics committee approval

The Maltepe University Clinical Research Ethics Committee approved the study (approval date: 2022; approval number: 2023/900/07), which was carried out in adherence to the Declaration of Helsinki II.

Statistics

Windows Statistical Package for Social Sciences (SPSS) version 22.0 (IBM SPSS Statistics, Armonk, NY) was used for all statistical calculations. Frequency, percentage, standard deviation, and mean values were used. Normality in numerical data was evaluated over skewness (±1.5). Independent sample t-test, one-way analysis of variance (ANOVA), Pearson correlation, and Spearman correlation were used. Pearson chi-square test and likelihood ratio were used in categorical data analysis and interpreted at a 95% confidence level. A p-value of <0.05 was considered statistically significant.

Limitations of the study

The following are some limitations of this study: retrospective design, small sample size, the lack of a control group, and not accounting for potential confounding variables that could influence biotin levels or laboratory parameters such as dietary habits, medication use, comorbidities, and other relevant clinical variables. There is a need for further research with more robust methodologies to draw reliable conclusions about the relationship between biotin levels and dermatological conditions.

## Results

Demographic data

There were 75 (71.4%) female and 30 (28.6%) male patients. The mean ages of the group were 32.68±16.47. Biotin levels were detected in 27 (25.7%) patients as deficient, in 48 (45.7%) patients as suboptimal, and in 30 (28.6%) patients as optimal (Table 1). There was no systemic disease in 73 patients (69.5%), 8.57% had hypothyroidism, 6.66% had cardiovascular disease, 3.8% had diabetes mellitus, 3.8% had depression, 2.86% had familial Mediterranean fever, 2.86% had insulin resistance, 1.9% had asthma, 0.95% had coronary artery disease, 0.95% had cerebrovascular disease, 0.95% had bipolar disorder, and 0.95% had malignancy.

**Table 1:** Demographic features and biotin levels

	n		%	
Gender				
Female	75		71.4	
Male	30		28.6	
Age groups				
<18	19		18.1	
18-50	68		64.8	
>50	18		17.1	
Biotin levels (ng/L)				
Deficiency (<200)	27		25.7	
Suboptimal (200-400)	48		45.7	
Optimal (>400)	30		28.6	
Total	105		100.0	
	Minimum	Maximum	Mean	Standard deviation
Age	5.00	75.00	32.68	16.47
Biotin levels	24.30	921.43	311.18	158.79
	n		%	
Comorbidities				
Hypothyroidism	9		8.57	
Hypertension	7		6.66	
Diabetes mellitus	4		3.8	
Depression	4		3.8	
Insulin resistance	3		2.86	
Familial Mediterranean fever	3		2.86	
Asthma	2		1.9	
Bipolar disorder	1		0.95	
Cerebrovascular disease	1		0.95	
Chronic urticaria	1		0.95	
Coronary artery disease	1		0.95	
Lung cancer	1		0.95	
Vitiligo	1		0.95	

Biotin levels

The mean biotin level was 311.18±158.79 in all patients. The average biotin levels in females were 302.48±158.92 and 332.94±159.06 in males. According to gender, there was no statistically significant difference in biotin levels (p=0.377). The average biotin levels were 256.88±106.5 in those under 18 years old, 307.4±154.28 in the 18-50 age group, and 382.81±199.12 in the group over 50 years. Although it was seen that it increased with age, it could not be considered statistically significant (p=0.05) (Table 2).

**Table 2:** Comparison of biotin levels by age and gender ^t^Independent sample t-test; ^F^one-way analysis of variance (ANOVA)

	x̄±s	t/F	p
Gender			
Female	302.48±158.92	-0.887^t^	0.377
Male	332.94±159.06		
Age			
<18	256.88±106.5	3.080^F^	0.050
18-50	307.4±154.28		
>50	382.81±199.12		

Biotin-measured dermatological diseases

The dermatological conditions for which biotin measurement has been made are as follows: 33 patients with telogen effluvium (31.4%), 19 with seborrheic dermatitis (18.1%), 18 with xerosis (17.1%), 17 with cold-induced hand dermatitis (16.2%), 15 with acne scars (13.2%), 12 with androgenetic alopecia (11.4%), four with keloid (3.8%), three with psoriasis (2.9%), two with keratosis pilaris (1.9%), one with alopecia areata (0.9%), one with dyshidrotic eczema (0.9%), one with erlotinib-induced skin rash (0.9%), one with rosacea (0.9%), one with urticaria (0.9%), one with a wart (0.9%), and one with vitiligo (0.9%) (Table 3).

**Table 3:** Reason for requesting biotin *Desire for more than one reason in a patient

	n	%	Biotin levels (ng/L) (x̄±s)
Telogen effluvium	33	31.4	323.02±144.21
Seborrheic dermatitis	19	18.1	309.41±119.8
Xerosis	18	17.1	290.19±142.23
Cold-induced hand dermatitis	17	16.2	273.57±140.19
Acne scars	15	14.3	281.19±182.89
Androgenetic alopecia	12	11.4	351.95±138.56
Keloids	4	3.8	322.96±321.95
Psoriasis	3	2.9	533.70±344.18
Keratosis pilaris	2	1.9	264.31±116.54
Alopecia areata	1	0.9	564.20
Dyshidrotic eczema	1	0.9	193.30
Erlotinib-induced skin rash	1	0.9	106.50
Rosacea	1	0.9	151.28
Urticaria	1	0.9	364.74
Wart	1	0.9	327.46
Vitiligo	1	0.9	533.05
All patients*	105	100.0	311.18±158.79

Comparison of biotin levels with laboratory values

There is a weak positive (r=0.201) relationship between biotin and creatinine. There is a weak positive (r=0.207) relationship between biotin and basophil. There is a weak negative (r=-0.216) relationship between mean platelet volume (MPV) and biotin. There is a moderate negative (r=-0.315) relationship between triglyceride and biotin. There is a moderate positive (r=0.314) relationship between total cholesterol/triglyceride and biotin (Table 4).

**Table 4:** Correlation coefficients between biotin levels and laboratory parameters *P<0.05 MCV, mean corpuscular volume; MCH, mean corpuscular hemoglobin; MCHC, mean corpuscular hemoglobin concentration; RDW-CV, red blood cell distribution width-coefficient of variation; HCT, hematocrit; PCT, plateletcrit; MPV, mean platelet volume; PDW, platelet distribution width; AST, aspartate aminotransferase; ALT, alanine aminotransferase; sp, Spearman’s rho coefficient

	Biotin		Biotin		Biotin
Leucocyte (%)	-0.087	Erythrocyte (10^3^/µl)	0.030	Creatinine (mg/dL)	0.201*
Neutrophils (%)	-0.041	HCT	0.076	AST (U/L)	-0.061_sp_
Lymphocytes (%)	-0.051	Hemoglobin (g/dL)	0.012	ALT (U/L)	0.089_sp_
Monocytes (%)	-0.004	MCV	0.091	Total cholesterol (mg/dL)	0.042
Eosinophils (%)	-0.009	MCH	-0.013	Triglyceride (mg/dL)	-0.315*
Basophils (%)	0.207*	MCHC	-0.178	Total cholesterol/triglyceride	0.314*
Neutrophils (/µl)	-0.079	RDW-CV	-0.064_sp_	Ferritin (ng/mL)	0.170
Lymphocytes (/µl)	-0.046	Platelet (/µl)	0.009	25-OH vitamin D (ng/mL)	0.095
Neutrophils/lymphocytes	-0.029	PCT	-0.149	Vitamin B12 (pg/mL)	0.086
Monocytes (/µl)	-0.122	PDW	0.153_sp_	Folic acid (ng/mL)	0.119
Eosinophils (/µl)	-0.122	MPV	-0.216*	Magnesium (mg/dL)	0.086
Basophils (/µl)	0.162	Fasting glucose (mg/dL)	-0.032	Zinc (μg/dL)	0.164

In the comparison of biotin levels to other vitamin and mineral levels, there was no statistically significant difference between any two variables (Table 5).

**Table 5:** Comparison between biotin levels and other vitamin or mineral deficiency t, independent sample t-test; F, one-way analysis of variance (ANOVA)

	n	x̄±s	F/t	p
25-OH vitamin D (ng/mL)				
Severe deficiency (1-10)	16	306.25±209.11	0.285	0.752
Moderate deficiency (10-30)	57	307.93±140.56		
Normal (over 30)	26	335.31±170.31		
Vitamin B12 (pg/mL)				
<187	7	306.36±137.79	0.810	0.448
187-883	90	305.72±162.13		
>883	4	409.87±148.35		
Folic acid (ng/mL)				
Deficient (<6)	35	287.27±172.47	-1.274	0.206
Normal (6-20)	59	331.61±157.44		
Magnesium (mg/dL)				
Deficient	2	197.9±245.51	1.220	0.305
Normal	39	348.33±168.48		
Excess	5	269.95±64.51		
Zinc (μg/dL)				
Deficient (<68)	4	323.83±63.29	0.065	0.937
Normal (68-115)	42	315.08±138.1		
Excess (>115)	12	331.27±158.13		

Other vitamin and mineral levels in dermatological conditions where biotin levels are evaluated most frequently were examined by making a table (Table 6).

**Table 6:** Evaluation of biotin and other vitamin or mineral deficiencies according to the dermatological diseases

	All patients		Seborrheic dermatitis		Telogen effluvium		Xerosis cutis		Cold-induced hand dermatitis		Acne scars	
	n	%	n	%	n	%	n	%	n	%	n	%
Biotin (ng/L)												
Deficient (<200)	27	25.7	4	21	7	21.2	5	27.8	7	41.2	4	26.7
Suboptimal (200-400)	48	45.7	9	47.4	16	48.5	9	50	7	41.2	8	53.3
Optimal (>400)	30	28.6	6	31.6	10	30.3	4	22.2	3	17.6	3	20
25-OH vitamin D (ng/mL)												
Serious deficiency (1-10)	16	16.2	0	0	3	9.7	7	43.7	4	25	1	7.1
Moderate deficiency (10-30)	57	57.5	14	77.8	18	58.1	7	43.7	11	68.75	8	57.1
Normal (>30)	26	26.3	4	22.2	10	32.2	2	12.5	1	6.25	5	35.7
Vitamin B12 (pg/mL)												
Deficient (<187)	7	6.9	1	5.9	2	6.7	1	9.1	1	6.7	1	9.1
Normal (187-883)	90	89.1	16	94.1	28	93.3	9	81.8	14	93.3	9	81.8
Excess (>883)	4	3.96	0	0	0	0	1	9.1	0	0	1	9.1
Folic acid (ng/mL)												
Deficient (<6)	35	37.2	9	47.4	10	33.3	7	38.9	6	46.2	9	69.2
Normal (6-20)	59	62.8	10	52.6	20	66.7	11	61.1	7	53.8	4	30.8
Magnesium (mg/dL)												
Deficient	2	4.3	0	0	2	11.1	0	0	0	0	0	0
Normal	39	84.8	8	88.9	14	77.8	5	71.4	5	83.3	4	100
Excess	5	10.9	1	11.1	2	11.1	2	28.6	1	16.7	0	0
Zinc (μg/dL)												
Deficient (<68)	4	6.9	0		0	0	2	20	1	12.5	0	0
Normal (68-115)	42	72.4	11	73.3	19	82.6	7	70	7	87.5	2	50
Excess (>115)	12	20.7	4	26.7	4	17.4	1	10	0	0	2	50

## Discussion

Biotin is widely known to be beneficial for the hair, nails, and skin, but there are only a few studies on the role of biotin in skin diseases. In dermatology, it is suggested that biotin may play a role in hair loss, brittle nails, seborrheic dermatitis, and the reduction of xerosis in patients using isotretinoin, but there is insufficient scientific evidence on this subject [4].

It is reported that biotin levels are not different according to gender [5]. In our study, we did not find a statistically significant difference between genders in biotin levels, which is consistent with the literature.

In a study examining age-related changes in the content of B group vitamins in the urine, blood, and liver of rats, it was found that biotin levels in the blood, liver, and urine were higher in aged rats compared to young rats [6]. Another study conducted on rats reported a significant increase in plasma biotin levels with aging, attributed to the rise in intestinal biotin transport [7]. Similarly, studies have reported an age-related increase in biotin levels in humans, although this change did not reach statistical significance [5]. Consistent with these findings, our study also observed an increase in biotin levels with advancing age. However, since the p-value was 0.05, we were unable to consider it statistically significant.

Biotin is one of the nutrients that are highly dependent on the production of intestinal bacteria [8]. There are changes in the intestinal microbiota with aging, the most notable feature being a change in the relative proportions of Firmicutes and Bacteroidetes. Older people have higher rates of Bacteroidetes, while younger adults have higher rates of Firmicutes [9]. Bacteroidetes are intestinal bacteria that play an important role in biotin synthesis [10]. A slight increase in biotin levels with aging may be associated with changes in gut microbiota. An increase in biotin-producing bacteria or a decrease in biotin-consuming bacteria may be responsible for this.

We found only one study in the literature that was similar to our study. In this study, the relationship between biotin status and some biochemical parameters in people over 65 years of age in Japan was investigated. Biotin levels were positively correlated with total cholesterol levels, while serum albumin, triiodothyronine, phosphate, and calcium levels were negatively correlated [5]. The biochemical parameters we compared in common with this study were total cholesterol, transaminases, white blood cells, red blood cells, hemoglobin, and hematocrit. They found a positive association between total cholesterol and biotin status, while we did not. Similarly, we also did not detect any relationship between biotin status and transaminases, white blood cells, red blood cells, hemoglobin, and hematocrit.

Among the hemogram parameters, we found only a weak positive correlation between the basophil ratio and biotin level in white blood cells. After our literature review, we thought that this relationship had no clinical significance.

In a study evaluating biotin deficiency in terms of mortality risk in kidney transplant recipients, biotin levels have been reported to be significantly higher in kidney transplant recipients compared to healthy controls [11]. It has been said that biotin may cause a prolonged half-life and high plasma concentration due to decreased biotin clearance in patients with renal failure [12]. However, we could not find a study in the literature examining creatinine levels and blood biotin levels. We found a weak positive relationship between biotin and creatinine. We think that impaired kidney function increases the level of biotin in the blood, but this needs to be investigated.

In a meta-analysis examining the effect of biotin usage on glycemic control and lipid profile in patients with type 2 diabetes mellitus conducted in 2022, it was reported that biotin treatment can reduce fasting blood glucose, total cholesterol, and triglyceride levels [13]. Also, in a study conducted with twins, it was reported that biotin levels were negatively correlated with triglyceride levels [14]. In the literature, we found a moderate negative correlation between biotin levels and triglyceride levels. We recommend checking biotin levels in the patients with high triglyceride levels.

In another study, the effects of biotin treatment on cholesterol, triglyceride, glucose, and insulin in diabetic and nondiabetic individuals were investigated. And it has been found that biotin treatment has no significant effect on cholesterol, glucose, and insulin but reduces hypertriglyceridemia [15]. Similar to these findings, we also found that biotin status was not associated with cholesterol and glucose levels in our patients, and there was a moderate negative correlation with triglyceride levels. Future studies should also consider the role that biotin deficiencies play in hypertriglyceridemia.

Mean platelet volume (MPV) has been shown to reflect platelet activity and is considered a useful predictor and prognostic biomarker of cardiovascular events. Increased MPV has been reported in cardiovascular diseases, cerebral palsy, respiratory diseases, chronic renal failure, intestinal diseases, rheumatoid diseases, diabetes, and various cancers [16]. Both animal and human biotin studies suggest a strong correlation between biotin and essential fatty acid (EFA) deficiencies. Thus, individuals consuming diets low in EFA and biotin may be at an increased risk of developing cardiovascular diseases. The relationship between biotin and glucose/insulin metabolism further implicates biotin as an essential nutrient for the prevention of cardiovascular diseases [17]. An unexpected association between MPV and serum triglyceride was found in one of the largest studies of MPV and dyslipidemia, examining 2,642 cases [18]. We found a weak negative (r=-0.216) relationship between MPV and biotin levels in our patients. The relationship between MPV and biotin levels may be secondary to the relationship between triglyceride levels and biotin. Based on these findings, we think that biotin may affect cardiovascular diseases, one of the systemic diseases.

The available literature regarding biotin levels or supplementation in dermatological conditions is limited. In our study, we primarily focused on evaluating biotin levels in the patients with seborrheic dermatitis, telogen effluvium, androgenic alopecia, xerosis cutis, cold-induced hand dermatitis, and acne scarring. Our aim was to investigate the potential association between biotin levels and other vitamin and mineral levels in these specific dermatological conditions. However, due to the limited number of cases, we were unable to conduct a statistical analysis to assess this relationship. Table 6 provides an overview of our findings in this regard.

Biotin levels were measured in the patients with acute or chronic telogen effluvium. There was no significant difference in serum biotin levels between cases and controls [2]. In a study in which serum biotin values of 541 females complaining of hair loss were measured, it was reported that seborrheic dermatitis-like dermatitis was observed in 35% of those with biotin deficiency, while none of those with optimal biotin levels had seborrheic dermatitis-like dermatitis [3].

Intravenous biotin was given to 25 infants with generalized seborrheic dermatitis in 1975, and it was reported that all patients recovered [19]. In the following years, no publications found that seborrheic dermatitis improved with intravenous biotin. In 1982, they conducted a placebo-controlled study by giving biotin treatment to patients with infantile flexural seborrheic dermatitis and reported that it did not change the course of seborrheic dermatitis [20].

Biotin is required for zinc homeostasis in the skin [1]. We thought that the coexistence of biotin deficiency and zinc deficiency might play a role in the emergence of dermatological conditions. Most of our patients had normal zinc levels, and we did not see any finding suggestive of such a relationship.

Oral biotin therapy has been reported to improve xerosis in children with biotin-deficient atopic dermatitis [21]. Biotin and biotinidase activity were evaluated in the patients receiving isotretinoin. It was found that there was no relationship between the severity of xerosis and the level of biotin and biotinidase activity [22]. While the rate of biotin deficiency was 25.7% in all our patients, similarly, biotin deficiency was present in 27.8% of patients with xerosis cutis.

It has been reported that there is a relationship between serum 25-OH vitamin D levels and stratum corneum hydration [23]. Vitamin D deficiency was significantly higher in the patients with xerosis cutis and cold-induced hand dermatitis (respectively, 87.5% and 93.75%). Therefore, we recommend checking vitamin D levels in those with dry skin and cold-induced hand eczema.

While the rate of folate deficiency was 37.2% in all of our patients, 69.2% of the patients with prominent acne scarring had folate deficiency. We suggest investigating the role of folate in scar development in acne. We did not find any study on the role of folate in scar development or scar development in acne. However, it has been reported that the use of isotretinoin can reduce serum folic acid levels [24]. Therefore, checking the folate levels of the patients who will be started on isotretinoin due to acne and supplementing with folate if it is deficient may help in reducing the development of scarring in acne.

One of our patients had a skin rash due to erlotinib, which she used for the treatment of lung cancer, and biotin deficiency (106 ng/mL) was detected. Biotin was administered to four patients with skin rash due to epidermal growth factor receptor tyrosine kinase inhibitors gefitinib and erlotinib, and skin eruptions decreased [25]. There were no reports of biotin levels in the patients receiving epidermal growth factor receptor tyrosine kinase inhibitors. We recommend that biotin levels be checked in the patients who are taking epidermal growth factor receptor tyrosine kinase inhibitors and have skin side effects.

## Conclusions

Biotin levels do not show a significant difference with gender but increase with age. Although a weak correlation was detected between hemogram parameters and biotin levels with basophil percentage and MPV values, we think that biotin did not significantly change hemogram parameters. The relationship between biotin levels and triglyceride levels is the most critical finding of our study. We recommend examining biotin levels in the patients with high triglyceride levels. In addition, although we have only one case, the biotin level was found to be low in the patient with skin rash due to erlotinib use. And patients taking epidermal growth factor receptor tyrosine kinase inhibitors deserve to have their biotin levels examined. Biotin supplementation is widely used. There is no consensus on biotin treatment and follow-up. We recommend that biotin supplementation should be made only in patients with deficiencies and that biotin levels should be measured in the follow-up.
